# Evaluation of the Efficacy and Safety of On-Demand Tadalafil Alone or Combined With Lidocaine Spray for Treating Patients With Comorbid Erectile Dysfunction and Premature Ejaculation: A Randomized Controlled Trial

**DOI:** 10.1080/19317611.2024.2420048

**Published:** 2024-11-10

**Authors:** Min Liu, Zi Xiang Deng, Xianxiang Zhu, ShiAn Hu

**Affiliations:** a Department of Reproductive Medicine, The First Affiliated Hospital of Gannan Medical University, Ganzhou City, China; b Department of Nephrology, The First Affiliated Hospital of Gannan Medical University, Ganzhou City, China; c Gannan Medical University, Gannan Medical College, Ganzhou, China

**Keywords:** Erectile dysfunction, premature ejaculation, tadalafil, lidocaine spray, randomized controlled trial

## Abstract

**Background:**

To evaluate the efficacy and safety of on-demand 20 mg tadalafil alone or combined with lidocaine spray for treating patients with comorbid erectile dysfunction (ED) and premature ejaculation (PE).

**Methods:**

This randomized controlled trial included 98 patients with comorbid ED and PE, randomly assigned to the experimental group (*n* = 50, tadalafil alone) or the active control group (*n* = 48, tadalafil combined with lidocaine spray). Patients took tadalafil 30 min before sexual intercourse, while the combination therapy group also used lidocaine spray 5 min before intercourse. After 12 weeks of treatment, changes in the International Index of Erectile Function (IIEF-5), Chinese Index of Premature Ejaculation (CIPE-5), Intravaginal Ejaculatory Latency Time (IELT), and Sexual Satisfaction Score (SSS) were evaluated.

**Results:**

Both groups showed significant improvement in IIEF-5 scores, but the difference between groups was not significant (*F* = 0.89, *p* = 0.45). The active control group showed significant improvement in CIPE-5 scores (*p* < 0.001), while the experimental group showed no significant change (*p* > 0.05). IELT in the active control group increased significantly from 25.7 s to 198.6 s (*Z* = 6.03, *p* < 0.001), while the experimental group showed no significant change (*Z* = 0.74, *p* = 0.46). SSS improved significantly in the active control group (*p* < 0.001) but not in the experimental group (*p* > 0.05). The incidence of adverse events was similar in both groups (14% vs 20.8%, *p* = 0.575).

**Conclusion:**

Tadalafil combined with lidocaine spray is superior to tadalafil alone in improving symptoms, prolonging IELT, and enhancing sexual satisfaction in patients with comorbid ED and PE, with comparable safety. This combination therapy provides a new approach for improving treatment strategies for patients with comorbid ED and PE. Large-scale, multi-center randomized controlled trials are needed to further validate these findings.

## Introduction

Erectile dysfunction (ED) is defined as the inability to achieve or maintain an erection sufficient for satisfactory sexual activity (Bekralas, 2022). The definition of premature ejaculation (PE) has evolved over time. In 2014, the International Society for Sexual Medicine (ISSM) defined lifelong PE as ejaculation that almost always occurs within 1 min, with an inability to delay ejaculation, accompanied by feelings of disappointment, frustration, and avoidance of intimate relationships (Gillman & Gillman, 2019). Acquired PE refers to individuals who previously had normal ejaculatory control but now ejaculate within approximately three minutes (Coskuner & Ozkan, 2022). Lifelong PE is typically caused by early sexual experiences, psychological factors (such as sexual abuse, negative body image, depression), or biological factors (such as hormonal imbalances and brain chemical abnormalities), while acquired PE is often triggered by psychological or biological factors after a period of normal sexual function (Martin-Tuite & Shindel, 2020).

There is a complex relationship between PE and ED, with a high comorbidity rate; PE patients have a 12.7-fold increased risk of ED, and vice versa (Porst et al., 2007). Pharmacological treatment is a common approach for PE (Sansone et al., 2023). Research indicates that PE is associated with increased penile sensitivity, making topical anesthetics an effective treatment option for PE, superior to placebo and oral medications (Liu et al., 2020; Shah et al., 2023). In treating ED, phosphodiesterase type 5 inhibitors (PDE5is) such as sildenafil and tadalafil are commonly used, with the latter being favored for its longer half-life and flexibility (von Büren et al., 2022). On-demand use of tadalafil has higher compliance compared to daily use, offering greater flexibility, lower psychological burden and cost, while maintaining therapeutic effect, making it more suitable for the needs of most ED patients (Zhou et al., 2019).

For patients with both ED and PE, there is controversy regarding the treatment sequence and combination medication. The American Urological Association (AUA) recommends treating ED first (Montague et al., 2005), while the European Association of Urology guidelines support using PE medications alone or in combination based on the latest data (Bai et al., 2015). Some studies have found tadalafil to be effective and well-tolerated in patients with comorbid PE and ED (El-Hamd, 2018; Gresser & Gleiter, 2002), but the role of PDE5is in treating PE remains controversial (Jannini et al., 2011).

Considering the onset and half-life of tadalafil and dapoxetine, tadalafil needs to be taken half an hour in advance, while dapoxetine should be taken one hour before sexual activity. Their effects are not fully synchronized, and combined use also has non-negligible side effects (Abu El-Hamd & Abdelhamed, 2018). In contrast, lidocaine spray acts quickly and can be used five minutes before sexual activity (Cai et al., 2022). Therefore, combining the long-term effects of tadalafil with the short-term action of lidocaine is theoretically superior to the combination of dapoxetine and long-acting drugs, improving flexibility and specificity while having lower side effects. Although there are studies on patients with comorbid ED and PE, there is currently no literature evaluating the on-demand combined use of 20 mg tadalafil and lidocaine spray. This study aims to assess the efficacy and safety of on-demand combined use of 20 mg tadalafil and lidocaine spray in treating ED and PE, with a three-month follow-up period.

## Materials and methods

This study is a randomized controlled clinical trial conducted from March to September 2023, targeting patients reporting erectile dysfunction and accompanying symptoms of lifelong/acquired premature ejaculation. The study was approved by the First Affiliated Hospital of Gannan Medical University (Approval No.: LLSC2024019), and all participants signed written informed consent. The study included couples who had regular sexual activity in the past six months. Detailed medical histories (including sexual history) were collected for each patient, and comprehensive physical examinations were performed. Eligible patients underwent a screening diagnostic period during which sexual frequency, IELT, sexual performance, and sexual satisfaction of both partners were recorded. Patients with heart failure, chronic prostatitis/chronic pelvic pain syndrome, paraplegia, depression, schizophrenia, those taking psychiatric medications, undergoing hemodialysis, with endocrine hormone abnormalities, epilepsy, and Parkinson’s disease were excluded.

**Figure 1.**
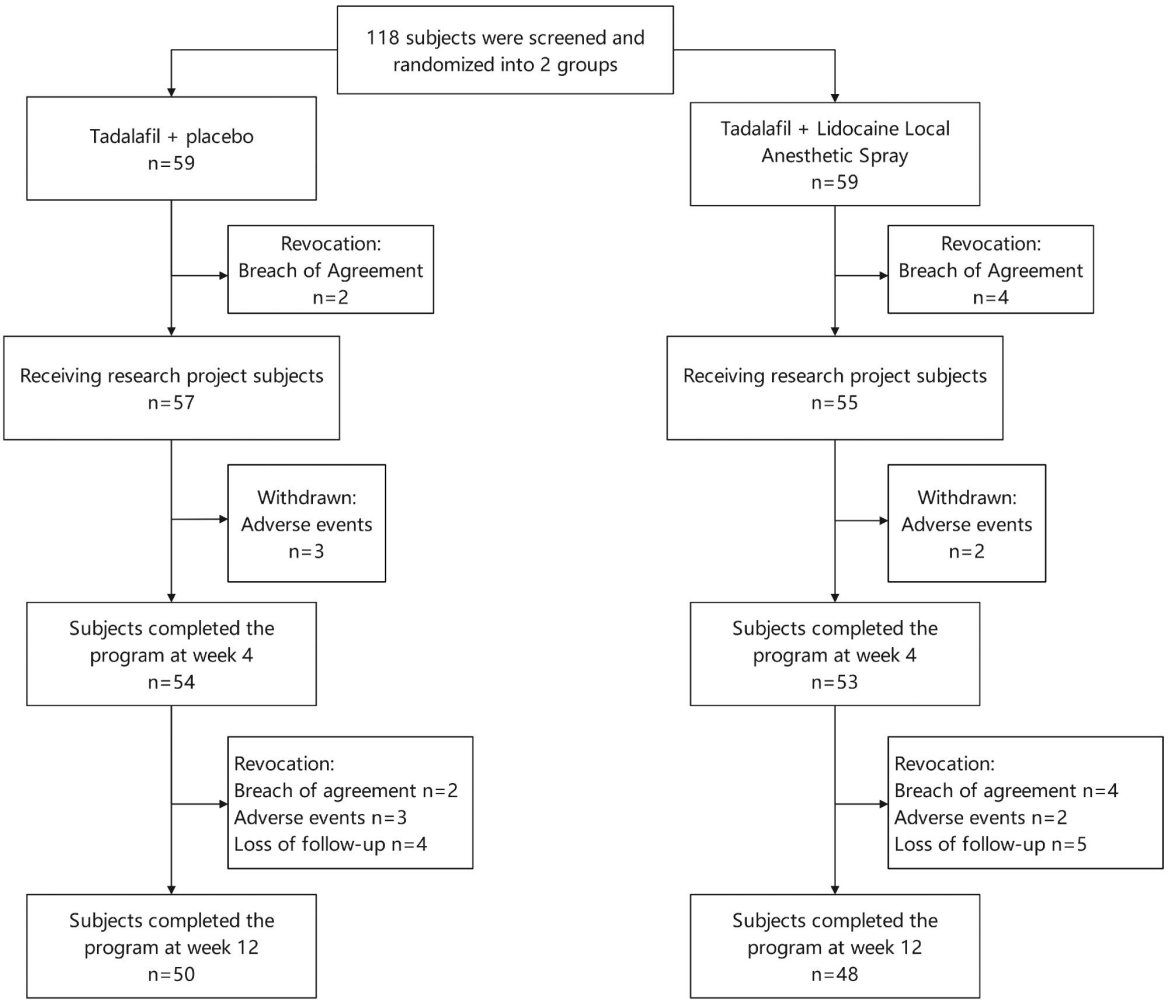
Clinical research flowchart.

This study followed the CONSORT (Consolidated Standards of Reporting Trials) principles for randomized controlled trials, employing a single-blind design where participants were unaware of the specific treatment they were receiving. Study team members were responsible for implementing blinding measures to avoid potential bias. The randomization sequence was generated using a computer-generated random number table, ensuring each subject had an equal chance of being assigned to either treatment group. Participants were randomly assigned to one of two groups after enrollment: tadalafil alone group (experimental group) or tadalafil combined with lidocaine spray group (active control group). A detailed study flow chart is shown in Figure 1. The allocation process was executed by research assistants without the participants’ knowledge, ensuring concealment of allocation. If subjects changed their treatment method or withdrew from the study during the research process, the reasons were recorded and reported.

To assess the degree of ED, this study used the Chinese version of the International Index of Erectile Function questionnaire. This questionnaire has been culturally and linguistically adapted and widely used in Chinese clinical and research settings for over 10 years to help evaluate and monitor male erectile function. The total IIEF-5 score ranges from 5 to 25, with scores below 21 considered indicative of ED (Rosen et al., 1997). PE was diagnosed using the CIPE-5, which covers five key areas: ejaculation control, sexual satisfaction, ejaculatory latency time, partner satisfaction, and sexual anxiety, with each question having five options ranging from “very poor” to “good” (Yuan et al., 2004). CIPE-5 classifies PE severity as mild (>13 points), moderate (10–13 points), and severe (5–9 points), providing a basis for evaluating treatment effectiveness. IELT is a common standard for assessing PE treatment efficacy, typically recorded using the stopwatch method (Althof et al., 2020; Castiglione et al., 2016). Additionally, SSS was used to evaluate personal or partner sexual satisfaction, reflecting quantitative indicators of sexual well-being, sexual function, and partner relationship quality (Ji et al., 2017; Kałużna et al., 2021).

The study used 10 mL spray bottles (JINGSIXUAN company, SKU:10089239522862) as carriers, transferring 5 mL of saline (Sichuan Kelun Pharmaceutical, H20083400) or 5 mL of 2% lidocaine solution (Shandong Hualu Pharmaceutical, H37022147) using syringes. The spray bottles were identical in appearance, and the liquids were colorless and odorless, conforming to CONSORT randomized control principles. The control group received saline spray, while the active control group received lidocaine spray, with bottles labeled with computer-generated random number tags.

Patients took 20 mg tadalafil orally (Eli Lilly Netherlands, H20170021) 30 min before intercourse (Eardley et al., 2002), and evenly sprayed the medication on the glans and penile surface when erect, 5 min before intercourse. At the end of the 12-week treatment, IIEF-5, CIPE-5, IELT, and SSS were reassessed, and side effects were recorded.

Patients were provided with usage guidelines before using the medication, including: recommendations to clean genitals after bathing, wait for at least 5 min after spraying for the drug to take effect. Wearing loose clothing was advised when walking at home to avoid the drug being rubbed off by fabric. Foreplay time was generally recommended not to exceed 15 min to avoid reducing drug efficacy. Ideal effect was described as the glans feeling numb but still having sensation, with a thickened or flattened feeling indicating drug effectiveness. Care was advised not to spray directly into the urethral opening. Overdosage could lead to numbness of the glans, potentially causing difficulty in ejaculation or even inability to ejaculate. Glans numbness was noted as temporary, naturally recovering after the drug effect wears off. Using condoms after spraying the glans was recommended to prevent the drug from being rubbed off and to avoid transferring the drug to the vagina. Most people have sexual intercourse once a week, generally not leading to drug resistance issues. Those using condoms were advised that washing was not necessary before intercourse, but to avoid condom breakage during intercourse. Those not using condoms were advised to wash before intercourse to prevent the drug from entering the vagina. These guidelines were made into portable cards for patients to follow the correct medication method.

This study employed various statistical methods for data analysis. Independent sample t-tests were used to compare differences in continuous variables between the experimental and control groups, such as age, BMI, marriage duration, etc. Paired *t*-tests were used for intra-group comparisons to evaluate differences before and after treatment, such as IIEF-5 and CIPE-5 scores, sexual satisfaction scores. One-way analysis of variance (ANOVA) was used for inter-group comparisons after treatment. Chi-square tests were used for comparing categorical variables, such as PE and DE classifications. Wilcoxon signed-rank tests were used for non-normally distributed variables, such as intra-group comparisons of IELT data. Mann-Whitney *U* tests were used for inter-group comparisons of IELT data. Fisher’s exact tests were used to compare the incidence of adverse reactions. All statistical analyses were performed using SPSS 25.0 software, with significance level set at *p* < 0.05.

## Results

This study recruited a total of 118 patients, of whom 98 (83.1%) completed the entire process. Twenty patients withdrew from the study due to reasons including: noncompliance with the study protocol (*n* = 6, such as insufficient frequency of sexual activity, travel, refusal to take medication), occurrence of side effects (*n* = 5), and loss to follow-up (*n* = 9). The study compared the baseline characteristics and pretreatment scores of the experimental group (*n =* 50) and the active control group (*n* = 48 patients) to evaluate the effects on PE and ED. Table 1 details the baseline characteristics and demographic information of the study population. After 12 weeks of treatment, 50 (84.7%) and 48 (81.4%) patients completed the study in the experimental group and active control group, respectively, with no statistically significant difference in completion rates between the two groups (*χ*^2^ = 0.235, *p* = 0.628). There were no significant differences between the two groups in all baseline characteristics (all *p* > 0.05), indicating that the randomization process was effective.

**Table 1.** Demographic and baseline characteristics of the study population.

Population characteristics and baseline	Experimental group *n* = 50	Active control group *n* = 48	*p*-Value
Age (years)	34.89 ± 6.10	32.83 ± 5.58	0.97
BMI	23.35 ± 4.28	21.6 ± 3.96	0.10
Duration of marriage (years)	4.03 ± 2.66	5.15 ± 3.05	0.15
Frequency of sexual intercourse/week	2.46 ± 0.78	2.16 ± 0.91	0.90
IELT (s)	27.4(13.0–43.3)	25.7(10.0–33.1)	0.33
ED+PE duration (years)	5.16 ± 2.13	4.61 ± 1.75	0.78
Pretreatment IIEF-5 score	11.76 ± 3.50	10.08 + 4.80	0.17
Pretreatment CIPE-5 score	9.9 ± 3.6	11.3 ± 4.3	0.87
Pretreatment sexual satisfaction			
Patient	2.25 ± 1.56	2.39 ± 1.32	0.30
Spouses	1.98 ± 1.44	2.15 ± 1.13	0.78
PE typing			
Primary	32	29	0.92
Secondary	18	19	
DE typing			
Primary	11	14	0.56
Secondary	39	34	
PE severity			
Mild	12	8	0.93
Moderate	24	28	
Severe	14	12	
DE severity			
Mild	11	10	0.84
Moderate	21	18	
Severe	18	20	

When comparing variables between the experimental group and the control group, independent samples *t*-tests were used for continuous variables (such as age, BMI, duration of marriage, etc.); chi-square tests were used for categorical variables (such as PE typing, DE typing, etc.). For IELT, which may not conform to a normal distribution, a non-parametric test method, the Mann-Whitney *U* test, was likely employed.

Table 2 shows the treatment effects in patients of different severity levels for both the experimental group and the active control group in this study, as evaluated by IIEF-5 and CIPE-5. The results indicate that both groups showed improvement in IIEF-5 scores. The active control group demonstrated significant improvement across all severity levels (*p* < 0.01), while the experimental group showed significant progress in moderate (*t* = 3.98, *p* = 0.001) and severe (*t* = 7.89, *p* < 0.001) patients. However, the difference between groups was not significant (*F* = 0.89, *p* = 0.45).

**Table 2.** Comparison of IIEF-5 and CIPE-5 scores before and after treatment in different severity levels for active control and experimental groups.

Indicator	Group	Severity level (n)	Before treatment	After treatment	Intra-group comparison	Inter-group comparison
IIEF-5	Active control group	Mild (10)	15.6 ± 3.8	20.7 ± 4.7	*t* = 3.21 *p* = 0.01	*F* = 0.89 *p* = 0.45
		Moderate (18)	11.4 ± 3.5	18.8 ± 4.5	*t* = 5.67 *p* < 0.001	
		Severe (20)	8.6 ± 2.9	14.4 ± 4.1	*t* = 5.23 *p* < 0.001	
	Experimental group	Mild (11)	17.1 ± 4.1	19.8 ± 4.9	*t* = 1.56 *p* = 0.15	
		Moderate (21)	13.4 ± 3.4	17.8 ± 3.7	*t* = 3.98 *p* = 0.001	
		Severe (18)	6.6 ± 2.0	13.8 ± 3.5	*t* = 7.89 *p* < 0.001	
CIPE-5	Active control group	Mild (8)	16.4 ± 4.2	19.7 ± 4.9	*t* = 1.68 *p* = 0.14	*F* = 7.23 *p* = 0.001
		Moderate (28)	12.2 ± 3.6	16.5 ± 4.1	*t* = 4.32 *p* < 0.001	
		Severe (12)	7.6 ± 2.0	15.5 ± 4.3	*t* = 5.23 *p* < 0.001	
	Experimental group	Mild (12)	15.2 ± 5.4	16.1 ± 5.2	*t* = 0.43 *p* = 0.67	
		Moderate (24)	12.5 ± 3.8	13.2 ± 4.9	*t* = 0.59 *p* = 0.56	
		Severe (14)	7.5 ± 2.8	7.8 ± 3.7	*t* = 0.25 *p* = 0.81	

Paired *t*-tests and one-way analysis of variance (ANOVA) were used as statistical methods. Paired *t*-tests were used for intra-group before-and-after comparisons, assessing changes in IIEF-5 and CIPE-5 scores for the same group of patients before and after treatment. One-way analysis of variance was used for inter-group comparisons to determine differences in various scores between the experimental group and the active control group after treatment.

Nevertheless, the CIPE-5 score results suggest that the active control group showed significant improvement in moderate to severe patients (*p* < 0.001), while the experimental group did not reach statistical significance across all severity levels (*p* > 0.05), leading to significant between-group differences (*F* = 7.23, *p* = 0.001). These findings suggest that both treatment methods are comparable in improving erectile function, but the active control group may have an advantage in alleviating premature ejaculation symptoms, especially for moderate to severe patients.

Table 3 presents the comparison of treatment effects on spouse and patient SSS (Sexual Satisfaction Score) and IELT (Intravaginal Ejaculatory Latency Time) between the experimental group and the active control group in this study. The results show that in the experimental group, the spouse SSS increased from 1.98 ± 1.44 before treatment to 2.05 ± 1.16 after treatment (*t* = 0.25, *p* = 0.80), while in the active control group, it significantly increased from 2.15 ± 1.13 to 5.84 ± 2.02 (*t* = 13.02, *p* < 0.001). For patient SSS, the experimental group increased from 2.25 ± 1.56 to 2.53 ± 1.68 (*t* = 1.28, *p* = 0.71), while the active control group significantly increased from 2.39 ± 1.32 to 6.20 ± 2.51 (*t* = 11.54, *p* < 0.001). IELT in the experimental group increased from 27.4(13.0–43.3) s to 30.2(11.0–43.3) seconds (*Z* = 0.74, *p* = 0.46), while in the active control group, it significantly increased from 25.7(10.0–33.1) seconds to 198.6(134-282) seconds (*Z* = 6.03, *p* < 0.001). Inter-group comparisons showed that the active control group was significantly superior to the experimental group in all indicators (*p* < 0.001), indicating that the treatment method in the active control group was more effective in improving couple SSS and prolonging IELT.

**Table 3.** Comparison of SSS and IELT before and after treatment in each group.

Indicator	Group	Before treatment	After treatment	Intra-group comparison	Intergroup comparison (post-treatment)
Spouse SSS	Experimental Group (*n* = 50)	1.98 ± 1.44	2.05 ± 1.16	*t* = 0.25 *p* = 0.80	*t* = 12.76 *p* < 0.001
	Active control group (*n* = 48)	2.15 ± 1.13	5.84 ± 2.02	*t* = 13.02 *p* < 0.001	
Patient SSS	Experimental Group (*n* = 50)	2.25 ± 1.56	2.53 ± 1.68	*t* = 1.28 *p* = 0.71	*t* = 9.23 *p* < 0.001
	Active control group (*n* = 48)	2.39 ± 1.32	6.20 ± 2.51	*t* = 11.54 *p* < 0.001	
IELT (秒)	Experimental Group (*n* = 50)	27.4 (13.0–43.3)	30.2 (11.0–43.3)	*Z* = 0.74 *p* = 0.46	*U* = 124 *p* < 0.001
	Active control group (*n* = 48)	25.7 (10.0-–33.1)	198.6 (134-–282)	*Z* = 6.03 *p* < 0.001	

Sexual Satisfaction Score (SSS) was measured using a 5-point scale. IELT data is presented as median (interquartile range) in seconds. Within-group comparison: SSS: Paired *t*-test, report *t*-value and *p*-value. IELT: Wilcoxon signed-rank test, report *Z*-value and *p*-value. Between-group comparison (post-treatment): SSS: Independent samples *t*-test, report *t*-value and *p*-value. IELT: Mann-Whitney *U* test, report *U*-value and *p*-value.

Table 4 shows the comparison of adverse events between the two groups. The observed adverse events included headache, flushing, nasal congestion, fatigue, nausea and vomiting, and dyspepsia. The results showed that the overall incidence of adverse events was not significantly different between the two groups (experimental group 7/50 vs. active control group 10/48, *p* = 0.575). This finding suggests that the two treatment regimens had similar safety and tolerability profiles.

**Table 4.** Comparison of adverse events between the experimental group and active control group.

Adverse reactions	Experimental group (*n* = 50)	Active control group (*n* = 48)	*p*值
Headaches	2	3	0.683
Flushing	1	1	1.000
Stuffy nose	1	0	1.000
Fatigue	1	2	0.620
Nausea and vomiting	1	2	0.620
Dyspepsia	1	2	0.620
In total	7	10	0.575

The *p*-value was calculated using Fisher’s exact test.

## Discussion

The study shows that PE and ED have a complex bidirectional relationship with significant comorbidity (Corona, 2022; Tsai et al., 2019). The controversy in the treatment of ED/PE comorbidity mainly focuses on the order of treatment and medication strategies. The AUA emphasizes comorbidity and suggests treating ED first (Sharlip, 2006). However, a study by S. Chia et al. pointed out that treating ED alone may not improve sexual satisfaction in patients with comorbidities, highlighting the necessity of also addressing PE treatment (Chia, 2002).

The latest meta-analysis shows that the combined use of PDE5is and SSRIs can significantly prolong IELT compared to using PDE5is alone, but the incidence of adverse effects is higher (Zhou et al., 2022). Dapoxetine is the first-line treatment for PE, usually recommended to be taken 1 h before sexual intercourse (Porst & Burri, 2019), while tadalafil is taken 30 min prior. Although dapoxetine has not reported serious adverse events, it has common side effects, and the long-term impact on reproductive function is still unclear (Assasa et al., 2019). It is worth noting that the discontinuation rate of dapoxetine treatment increases over time, with 80% of patients stopping within 6 months (Park et al., 2017).

The advantages of lidocaine spray in the treatment of PE include rapid IELT prolongation, high local safety, low risk of tachyphylaxis with intermittent use, and convenience of over-the-counter formulation (Abu El-Hamd, 2021). In theory, the short-term-long-term combination of lidocaine and tadalafil should be superior to the long-term-long-term combination of dapoxetine and tadalafil, and should significantly improve IELT and spousal SSS. The results of this study confirm this view.

This study was a randomized controlled clinical trial that evaluated the efficacy and safety of on-demand tadalafil versus the combination of tadalafil and lidocaine spray in the treatment of patients with ED and PE comorbidity. The results showed that both groups had significant improvements in IIEF-5 scores, but only the combination therapy group showed significant improvements in CIPE-5 scores, IELT, and SSS. Patients receiving tadalafil monotherapy did not show significant changes in these indicators, indicating that monotherapy has limited efficacy for ED/PE comorbid patients. Furthermore, the incidence of adverse events was similar between the two groups, and the combination therapy did not increase additional risks.

The study has certain limitations, including a small sample size (*n* = 98) that may reduce the statistical power, a 12-week follow-up period that may be insufficient to observe some long-term effects or side effects, the single-center nature that may affect the generalizability of the results, and the subjective nature of SSS and IELT data that may introduce bias. Additionally, the exclusion criteria may have led to selection bias, such as excluding patients with heart failure or chronic prostatitis/chronic pelvic pain syndrome, which are themselves causes of erectile dysfunction.

Based on the study results and limitations, we propose the following hypotheses for future research verification: (1) The combination of tadalafil and lidocaine spray can more significantly improve the symptoms of ED and PE comorbid patients; (2) Combination therapy can significantly prolong IELT and improve SSS; (3) The combination therapy is comparable to tadalafil monotherapy in terms of safety and tolerability. To further validate these hypotheses, we suggest future research: (1) Conduct large-scale multicenter randomized controlled trials; (2) Adopt more specialized efficacy assessment tools, such as the Premature Ejaculation-Specific Questionnaire (PESQ); (3) Use geometric mean analysis for IELT data. These improvements will help comprehensively evaluate the efficacy, reliability, and wide applicability of the combination therapy, and further optimize the treatment strategy for patients with ED and PE comorbidity.
